# “Unmasking the Uncommon”: A case series of multi-drug resistant Elizabethkingia meningoseptica causing late-onset sepsis and meningitis in preterm neonates

**DOI:** 10.12688/f1000research.158137.2

**Published:** 2025-02-06

**Authors:** Prajnha U.P., Anisha Maria Fernandes, Suchitra Shenoy M., Sinchana Bhat

**Affiliations:** 1 Department of Microbiology, Kasturba Medical College, Mangalore,, Manipal Academy of Higher Education, Manipal, Karnataka, 576104, India; 2 Regional Advanced Paediatric Care Centre, Government Wenlock Hospital, Mangalore, Manipal Academy of Higher Education, Manipal, Karnataka, 576104, India

**Keywords:** Elizabethkingia meningoseptica, late-onset, sepsis, meningitis, Multi-drug resistance

## Abstract

*Elizabethkingia meningoseptica* is an uncommon nosocomial pathogen that causes meningitis, pneumonia, and sepsis in neonates and in immunocompromised individuals. It exhibits resistance to many commonly employed first-line antibiotics used to treat gram-negative pathogens. Herein, we present three cases of late-onset sepsis with multi-drug resistant (MDR) *Elizabethkingia meningoseptica* in high-risk neonates.

Case 1 was a one-day-old preterm low-birth-weight infant who presented with respiratory distress syndrome and septic shock. The patient was intubated and administered empirical broad-spectrum antibiotics and antifungal agents. Blood culture grew *Candida krusei,* hence Amphotericin B was initiated. Repeat blood culture on day 27 showed gram-negative bacilli, identified as *Elizabethkingia meningoseptica* by MALDI-TOF *.* Antibiotic susceptibility testing (AST) revealed resistance to Piperacillin/Tazobactam, but sensitivity to Vancomycin, Levofloxacin, and Minocycline. IV Vancomycin was administered, which resulted in clinical improvement and negative blood culture results. Case 2 was an eleven-day-old preterm, low-birth-weight baby who presented with fever. Initial investigations revealed normal complete blood counts (CBC) parameters and elevated CRP levels. Blood and CSF cultures isolated *Elizabethkingia meningoseptica* with a similar AST pattern. Intravenous Ciprofloxacin was initiated with clinical improvement and negative follow-up blood cultures. Case 3 was a one-day-old preterm baby, appropriate-to-gestational age, presenting with respiratory distress syndrome. The infant was intubated and started on inotropic support and intravenous antibiotics. Blood cultures on day 4 showed *Elizabethkingia meningoseptica* and Vancomycin was started. Follow-up cultures on days 6 and 14 grew *Acinetobacter baumannii.* A combination of Levofloxacin and Colistin was added, and blood cultures were negative after seven days, with clinical improvement.

*Elizabethkingia meningoseptica* is a significant cause of hospital-acquired infections, especially in Neonatal Intensive Care Unit (NICU), leading to outbreaks. Clinicians must have a high degree of suspicion of *E. meningoseptica* for gram-negative bacilli causing sepsis and meningitis in high-risk patients. Recent technological advances have enabled accurate speciation to guide therapy and reduce morbidity and mortality rates.

## Introduction

*Elizabethkingia meningoseptica* is an uncommon cause of infection that mostly affects immunocompromised individuals. ^1^ In neonates, premature birth weight and very low birth weight are major risk factors. *E. meningoseptica* infections present as early onset sepsis and meningitis with a high mortality rate. Reports of late-onset sepsis and meningitis are rare. ^2^ *E. meningoseptica* is found in both natural and hospital environments, and is isolated from contaminated water supplies, equipment surfaces and tubing, infant formulas, and IV solutions. ^3, 4^ Its ability to form biofilms allows it to persist in hospital environments, with outbreaks being reported, especially in Neonatal Intensive Care Units (NICU). ^5^ Its intrinsic resistance and recent reports of multi-drug resistant strains (MDR) are a cause for growing concern. ^1^ In the absence of established guidelines for antibiotic susceptibility testing (AST) and lack of evidence-based treatment regimens, treatment failures are common with negative patient outcomes. ^1^

We present three cases of late-onset sepsis in high-risk neonates born at a district government hospital and admitted to the NICU simultaneously. All had a similar MDR AST profile but were successfully treated.

## Case report

### Case 1

A 1-day-old preterm baby (32 weeks + 1 day) with low birth weight (1.42 kg), born via emergency LSCS, presented with respiratory distress syndrome and septic shock. The neonate was transferred to the NICU, intubated, started on inotropic support, and administered intravenous antibiotics (cefotaxime and gentamicin) and antifungals (fluconazole). On examination, the patient’s temperature was 36.5°C, pulse rate was 180/min, respiratory rate was 50/min, bilateral crepitations, and blood pressure was 40/24 mmHg. Results of cardiovascular and abdominal examinations were normal. On central nervous system examination, the child appeared dull.

The complete blood count (CBC) was within normal limits. Blood collected for culture in a pediatric BD BACTEC ^TM^ (peds plus/F) bottle showed no growth. Serial counts showed a worsening trend, with increased CRP levels (23 mg/dL). On day 4, blood culture showed growth of *Candida krusei* with sensitivity to Voriconazole, Caspofungin and Amphotericin B. Voriconazole was initiated. Follow-up blood cultures sent on days 8 and 19 of admission showed persistence of *Candida krusei.* Hence, the treatment was changed to Amphotericin B.

A follow-up blood culture on day 27 of admission flagged as positive within 24 h. Gram staining showed gram-negative bacilli, and culture yielded aerobic, 1 to 2 mm smooth, circular, greyish white non-hemolytic colonies on blood agar, and non-lactose fermenting semitranslucent colonies on MacConkey agar. The isolate was identified as *Elizabethkingia meningoseptica* by matrix-assisted laser desorption ionization time-of-flight mass spectrometry (MALDI-TOF MS) (Biomerieux ^TM^) and antibiotic susceptibility testing (AST) performed using VITEK 2 Compact (Biomerieux ^TM^). Vancomycin susceptibility was tested by using an E-strip. The isolate was sensitive to Vancomycin, Levofloxacin, Minocycline, and resistant to Piperacillin/Tazobactam and Trimethoprim-sulfamethoxazole (TMP-SMX) Susceptibility was determined using the CLSI breakpoints for non-Enterobacterales except for Vancomycin, where MICs of <4μg/mL were considered susceptible. Antibiotics were changed IV Vancomycin 22 mg IV BD for 6 days. The child showed clinical signs of improvement and negative blood culture results.

### Case 2

An 11-day-old preterm (34 weeks + 2 days), low birth weight (1.86 kg) male baby delivered by normal vaginal delivery presented with fever. On admission, the child had a temperature of 100 °F. Neurosonogram results were normal. CBC, liver, and kidney function parameters were within normal limits; however, the CRP level was elevated (116 mg/dL). Blood cultures were collected in pediatric BACTEC bottles. The neonate was administered Piperacillin and Amikacin empirically.

The blood culture bottle flagged positive at 24 hours and showed short gram-negative bacilli. The organism was identified as *Elizabethkingia meningoseptica* with a sensitivity pattern identical to that of the first case. On day 8 of admission, as there was no clinical improvement and the CRP level was still elevated (131 mg/dL), lumbar puncture was performed. CSF cytology showed neutrophilic pleocytosis with increased protein (442 mg/dl) and decreased glucose (2 mg/dl) levels. CSF culture yielded *E. meningoseptica* with the same sensitivity pattern. The antibiotic was changed to intravenous Ciprofloxacin (20 mg TID). A follow-up blood culture on day 12 of admission showed no growth, and the markers of sepsis were negative. The patient showed clinical improvement.

### Case 3

A 1-day-old preterm baby (28 weeks + 2 day), appropriate for gestational age (970 g), born via emergency LSCS, presented with respiratory distress syndrome. The neonate was transferred to the NICU, intubated, and started on inotropic support and intravenous antibiotics (cefotaxime and amikacin).

On examination, the patient’s temperature was 36.5°C, pulse rate was 146/min, respiratory rate was 64/min, with bilateral crepitations. Results of cardiovascular and abdominal examinations were normal. On central nervous system examination, the child appeared dull. CBC was within the normal limits. Blood cultures showed no growth. Serial counts showed a worsening trend.

Blood culture collected on day 8 yielded *Elizabethkingia meningoseptica* within 36 h with the same AST pattern as the previous cases. Inj. Vancomycin (15 mg BD) was administered for 6 days. Follow-up blood cultures yielded pan-drug-resistant *Acinetobacter baumannii.* Inj Meropenem 20 mg BD, and IV Colistin 1.5 lakh IU/day in two divided doses was administered for 14 days, after which blood culture showed no growth, and the child showed clinical signs of improvement.

As all 3 cases were admitted within a few weeks of each other, environmental screening was done by water sampling and surface sampling from bed railings, sinks and faucets within the NICU. *E. meningoseptica* was not isolated from any of these sources.

## Discussion

*Elizabethkingia meningoseptica* is an emerging nosocomial pathogen widespread in natural environments. It can persist under harsh conditions in healthcare facilities and has been isolated from hospital water supplies and critical care equipment such as mechanical ventilators and indwelling catheters, resulting in outbreaks. ^3, 4, 6^ In neonates, *E. meningoseptica* infection typically manifests as either early onset sepsis or meningitis. Predisposing factors in neonates include prematurity, very low birth weight, central venous catheters, immunosuppression, and prolonged and prior exposure to higher antibiotic concentrations. ^1, 3^ Neurological complications are common in 30.4% of survivors who develop hydrocephalus and 6.5% experience varying degrees of hearing loss. ^2, 3^

Recent reports show a notable increase in *E. meningoseptica* infections, possibly due to advanced automated methods. ^3^ *E. meningoseptica* is resistant to a wide range of antimicrobials including most β-lactams (including cephalosporins and carbapenems), as well as in combination with β-lactamase inhibitors (except for piperacillin-tazobactam), macrolides, tetracyclines, polymyxins, chloramphenicol, and aminoglycosides. ^1, 3, 7^ Thus, most empirical treatments for gram-negative infections are ineffective. Global reports show a geographical variability in susceptibilities to fluoroquinolones, piperacillin/ tazobactam and TMP-SMX. Recent literature from USA ^8^ and Australia ^7^ have reported up to 89% susceptibility and 75% clinical success with piperacillin/tazobactam, unlike our isolates which were 100% resistant. Similar to global studies, our isolates expressed susceptibility to Levofloxacin and Minocycline. ^7^

Uniquely, susceptibility to antibiotics used to treat gram-positive infections such as Vancomycin, Clindamycin and Rifampicin has been reported in *Elizabethkingia* spp. Initially, susceptibility to vancomycin was demonstrated by disk diffusion, using the CLSI interpretative criteria for *Staphylococcus* spp. Discrepancies recognized in results of susceptibility testing for vancomycin in *E. meningoseptica* by disk diffusion and detection of MIC breakpoints by broth microdilution or agar dilution, resulted in the former method being abandoned. While lacking interpretative breakpoints in CLSI and EUCAST, treatment failures are seen with elevated MICs of ≥4μg/mL and isolates are considered non-susceptible. ^7^ Though initially advocated as the mainstay agent in the treatment of invasive *E. meningoseptica* infections, increasing reports of therapeutic failures are alarming. ^9^ There is a high prevalence of VanW gene in *Elizabethkingia* isolates from Australia and South East Asia, conferring a VanB type phenotype as seen in Gram positive organisms. ^10^ The wide variation in susceptibility highlights the need for MIC profiling.

The potential for multi-drug resistance and the absence of established guidelines for antibiotic susceptibility testing make management even more challenging. While some studies advocate a combination of minocycline and TMP-SMX with isolates demonstrating 90% susceptibility to TMP-SXM, ^8^ we noted our cluster of isolates to be resistant to TMP-SMX. Our patients showed good clinical outcomes with monotherapy of vancomycin in 2 cases and ciprofloxacin in 1 case. Fluoroquinolone monotherapy has proven to be effective in *E. meningoseptica* infections, however combination therapy is preferred to avoid high-level resistance developing due to single-step mutations. ^7, 10^ Vancomycin alone has been reported to have a high mortality (78%) in the management of neonatal meningitis. ^9^ Based on the available data, a combination of vancomycin and ciprofloxacin, linezolid, or rifampicin has demonstrated good clinical efficacy in the treatment of *E. meningoseptica* meningitis and bacteremia. ^1, 3^ The use of rifampicin-containing therapies may be curbed due to a paucity of data in effective management of Gram-negative infections, ^10^ as well as reserved in counties endemic with tuberculosis.

Attempts must be made to trace the source of the infection and to take stringent steps to prevent the transmission of this infection. Recommendations for environmental screening include water sampling and environmental swabs from sinks, faucets, patient beds, tubings and devices. ^6^ Infection prevention recommendations include consistently using alcohol-based hand rubs following hand washing and using sterile water to change diapers. It is also important to periodically repair, clean, super chlorinate, and replace sink taps. Continuous training is essential to reinforce proper hand hygiene practices and contact precautions for hospital personnel. ^11^

There are a few reports of *E. meningoseptica* causing late-onset sepsis and meningitis. ^1, 2^ Interestingly, all of our patients had late-onset sepsis and meningitis. Cases 1 and 3 presented with bacteremia, whereas case 2 showed meningitis. Prematurity was a common risk factor for all neonates. Additionally, cases 1 and 3 had other predisposing factors such as the use of central venous catheters and prolonged and prior exposure to higher antibiotic concentrations. Timely identification of cultures allows for prompt and tailored therapy with good patient outcomes. Long-term follow-up is required to assess the potential neurological complications. Neonates were admitted to the NICU during the same period. We hypothesized that our cases were nosocomial infections; however, environmental screening failed to identify the source of infection. Due to lack of funds, sequencing was not performed to determine the epidemiological relationship of the strains.

The presence of gram-negative bacilli causing bacteremia or meningitis in neonates with risk factors must alert clinicians to *E. meningoseptica.* Prompt and appropriate antibiotic therapy is key to curbing long-term morbidity and mortality.

## Ethics and consent

This study was approved by the Institutional Ethics Committee (Kasturba Medical College, Mangalore), Reg No. ECR/541/Inst/KA/2014/RR-20, DHR Reg. No. EC/NEW/INST/2020/742. Approval was given on 20.12.2023 with protocol number IEC KMC MLR-12/2023/513.

Written informed consent for publication of clinical details and/or clinical images was obtained from the parents of the patients.

## Data Availability

No data are associated with this article.
