# Supplementary material for: “Unmasking the Uncommon”: A case series of multi-drug resistant Elizabethkingia meningoseptica causing late-onset sepsis and meningitis in preterm neonates
Source: F1000Res. 2025 Feb 6;13:1367. Originally published 2024 Nov 15. [Version 2] doi: 10.12688/f1000research.158137.2 (PMC11814954; doi:10.12688/f1000research.158137.2)
Supplement: Peer review history

# Reviewer report: Reviewer response for version 2

**DOI:** 10.5256/f1000research.177523.r364980

**Published:** 2025-02-11

**Authors:** Mark Fahmy

**Affiliations:** 1 The University of Queensland, Saint Lucia, Queensland, Australia

Thank you to the authors for considering the comments made. The additional details are helpful in describing this rare pathogen and discussion of treatment strategies is well rounded.

Are enough details provided of any physical examination and diagnostic tests, treatment given and outcomes?

Partly

Is the background of the cases’ history and progression described in sufficient detail?

Yes

Is sufficient discussion included of the importance of the findings and their relevance to future understanding of disease processes, diagnosis or treatment?

Partly

Is the conclusion balanced and justified on the basis of the findings?

Partly

Reviewer Expertise:

Published a case series of Elizabethkingia BSI in adults.

I confirm that I have read this submission and believe that I have an appropriate level of expertise to confirm that it is of an acceptable scientific standard.

# Reviewer report: Reviewer response for version 1

**DOI:** 10.5256/f1000research.173690.r357608

**Published:** 2025-01-22

**Authors:** Mark Fahmy

**Affiliations:** 1 The University of Queensland, Saint Lucia, Queensland, Australia

The authors have written an interesting summary of three cases of *Elizabethkingia meningioseptica *BSI in pre-term infants. 

**Major comments:**

 In the discussion section **"wide range of antimicrobials including β-lactams..."**

 - would argue that some strains retain susceptibility to piperacillin - tazobactam (accepting none in this series were susceptible, which itself is interesting and worth discussing/evaluating in the context of existing literature). However, other published literature has reported susceptibility and clinical success with piperacillin-tazobactam 

 (e.g. Burnard D et al. 2020 (Ref 1)

 Comba IY, et al. 2022 (Ref 2)

 In the discussion section **" Uniquely, it is susceptible to antibiotics used to treat gram-positive infections such as Vancomycin, Clindamycin and Rifampicin..."**

 I think that the role of vancomycin and rifampicin for treatment of *Elizabethkingia* BSI is more uncertain than this discussion suggests, and while it might be reasonable to consider use in severe infections/as part of a multi-drug regimen in complex infections, there is a dearth of evidence suggesting the strong conclusion made by the authors **" a combination of vancomycin and ciprofloxacin, linezolid, or rifampicin has demonstrated high cure rates in the treatment of *E. meningoseptica* meningitis and bacteremia"**

 Further discussion about the ambiguities and gaps in the literature regarding these therapies for *Elizabethkingia* would allow for more nuanced recommendations. 

 In particular, it can be noted that

 1) In adult patients, there have been documented rates of failure with vancomycin therapy as details here:

 Jean SS, et al., 2017 (Ref 3)

 2) There is a high prevalence of VanW gene in *Elizabethkingia* isolates in Australia (and this has been demonstrated elsewhere as well). This gene has also been found to confer a VanB type phenotype in other organisms. 

 Stewart AG, et al., 2023 (Ref 4)

 3) There is overall limited data for the use of rifampicin in gram negative infections. 

 Drapeau CM, et al., 2010 (Ref 5)

**Minor comments:**

 In case 1 (and subsequent cases) susceptibility testing is described as use of VITEK-2 + E-test for susceptibility testing **. "antibiotic susceptibility testing (AST) performed using VITEK 2 Compact (Biomerieux ^TM^). Vancomycin susceptibility was tested by using an E-strip."**

 There could be better description of the interpretive criteria used to determine susceptibility. In particular, what breakpoints were used- I assume CLSI non-enterobacterales breakpoints for more traditional agents (e.g. piperacillin, levofloxacin) but there would not be calibrated breakpoints for vancomycin- reporting what the MIC result would be more accurate. Minocycline sensitivity can be inferred from doxycycline or tetracycline susceptibility- was this done or was a minocycline disc/etest performed?  

 I think the comments about control of environmental sources is very interesting. **" It is also important to periodically repair, clean, super chlorinate, and replace sink taps"** and **"We hypothesized that our cases were nosocomial infections; however, environmental screening failed to identify the source of infection."**

 I agree that a nosocomial infection is likely if all three cases were acquired around the same time - it may be worth mentioning that more clearly in the case histories to emphasize this point.

 However some additional information may be interesting and make the discussion more applicable to a wider audience-

 1) Was sequencing considered or done on the isolates from the patients to see if there was close relationship and potentially establish common epidemiological link?

 2) How was environmental sampling done- e.g. what was sampled, what methods were used to try and isolate *Elizabethkingia* sp.

 There are a variety of published methods for environmental sampling/isolation, none of them specific for *Elizabethkingia* > our laboratory uses these guidelines for sampling and isolation ( https://www.health.vic.gov.au/infectious-diseases/victorian-guideline-on-environmental-sampling-for-cpe)

 Otherwise the authors have prepared an interesting summary of three cases of this rare but important pathogen, including a good description of a variety of risk factors and clinical outcomes.

Are enough details provided of any physical examination and diagnostic tests, treatment given and outcomes?

Partly

Is the background of the cases’ history and progression described in sufficient detail?

Yes

Is sufficient discussion included of the importance of the findings and their relevance to future understanding of disease processes, diagnosis or treatment?

Partly

Is the conclusion balanced and justified on the basis of the findings?

Partly

Reviewer Expertise:

Published a case series of Elizabethkingia BSI in adults.

I confirm that I have read this submission and believe that I have an appropriate level of expertise to confirm that it is of an acceptable scientific standard, however I have significant reservations, as outlined above.

## References

[1] : Comparative Genomics and Antimicrobial Resistance Profiling of Elizabethkingia Isolates Reveal Nosocomial Transmission and In Vitro Susceptibility to Fluoroquinolones, Tetracyclines, and Trimethoprim-Sulfamethoxazole. *J Clin Microbiol* .2020;58(9) : 10.1128/JCM.00730-20 10.1128/JCM.00730-20 32580952 PMC7448627

[2] : Antimicrobial Susceptibility of Elizabethkingia Species: Report from a Reference Laboratory. *J Clin Microbiol* .2022;60(6) : 10.1128/jcm.02541-21 e0254121 10.1128/jcm.02541-21 35510517 PMC9199404

[3] : Role of vancomycin in the treatment of bacteraemia and meningitis caused by Elizabethkingia meningoseptica. *Int J Antimicrob Agents* .2017;50(4) : 10.1016/j.ijantimicag.2017.06.021 507-511 10.1016/j.ijantimicag.2017.06.021 28705672

[4] : Treatment Failure Due to Adaptive Resistance Mechanisms in a Severe and Complicated Bloodstream Infection Due to Elizabethkingia meningoseptica. *Microb Drug Resist* .2023;29(4) : 10.1089/mdr.2022.0179 145-149 10.1089/mdr.2022.0179 36827594

[5] : Rifampicin combined regimens for gram-negative infections: data from the literature. *Int J Antimicrob Agents* .2010;35(1) : 10.1016/j.ijantimicag.2009.08.011 39-44 10.1016/j.ijantimicag.2009.08.011 19815392

# Response

**Published:** 2025-01-25

**Authors:** Anisha Maria Fernandes

**Affiliations:** Microbiology, Manipal Academy of Higher Education, Manipal, Karnataka, India

The authors thank Dr. Fahmy for his in-depth review and suggestions.

 We have tried to address all the comments including expanding the discussions on suseptibility testing and interpretative criteria used, global patterns of susceptibility and therapeutic recommendations, as well as environmental screening methods used.

 Regarding susceptibility testing for Minocycline, the MIC testing was done by the VITEK 2 Compact (Biomerieux ^TM^).

# Reviewer report: Reviewer response for version 1

**DOI:** 10.5256/f1000research.173690.r341910

**Published:** 2024-11-25

**Authors:** Godwin Justus Wilson

**Affiliations:** 1 Hamad Medical Corporation, Weil Cornell Medical College, Ar-Rayyan, Qatar

This article provides an insight into *Elizabethkingia meningoseptica* infections, particularly in neonates, emphasizing their rare occurrence but severe implications, especially in immunocompromised or premature infants. The case studies presented offer valuable real-world examples of late-onset sepsis and meningitis caused by this pathogen, highlighting its multi-drug-resistant (MDR) nature and the challenges clinicians face due to the lack of standardized treatment guidelines.

 Overall, the article provides valuable clinical insights and emphasizes the need for prompt detection, tailored treatment, and robust infection control to improve outcomes for neonates affected by this challenging pathogen.

Are enough details provided of any physical examination and diagnostic tests, treatment given and outcomes?

Yes

Is the background of the cases’ history and progression described in sufficient detail?

Yes

Is sufficient discussion included of the importance of the findings and their relevance to future understanding of disease processes, diagnosis or treatment?

Yes

Is the conclusion balanced and justified on the basis of the findings?

Yes

Reviewer Expertise:

Antimicrobial resistance , Antimicrobial Stewardship, Clinical Informatics

I confirm that I have read this submission and believe that I have an appropriate level of expertise to confirm that it is of an acceptable scientific standard.
